# Pulsed electric field (PEF)–processed 6-shogaol-rich ginger extract protects β-Thalassemic red blood cells from iron-induced oxidative stress and hemolysis

**DOI:** 10.1371/journal.pone.0332386

**Published:** 2025-09-12

**Authors:** Hataichanok Chuljerm, Narisara Paradee, Kornvipa Settakorn, Somdet Srichairatanakool, Artit Yawootti, Kanokwan Kulprachakarn, Wason Parklak, Pimpisid Koonyosying

**Affiliations:** 1 School of Health Sciences Research, Research Institute for Health Sciences, Chiang Mai University, Chiang Mai, Thailand; 2 Research Center for Non-Infectious Diseases and Environmental Health, Research Institute for Health Sciences, Chiang Mai University, Chiang Mai, Thailand; 3 Department of Biochemistry, Faculty of Medicine Chiang Mai University, Chiang Mai, Thailand; 4 Clinical Research Center for Food and Herbal Product Trials and Development (CR-FAH), Faculty of Medicine, Chiang Mai University, Chiang Mai, Thailand; 5 Department of Pharmacology, Faculty of Medicine, Chiang Mai University, Chiang Mai, Thailand; 6 Faculty of Engineering, Rajamangala University of Technology Lanna, Chiang Mai, Thailand; Università degli Studi di Milano, ITALY

## Abstract

β- thalassemia is the genetic blood disorder characterized by ineffective beta-globin chain synthesis. The increased iron absorption and frequently blood transfusion result in iron overload and the production of reactive oxygen species (ROS). ROS causes lipid peroxidation and membrane damage in red blood cells (RBCs), culminating in hemolysis and anemia. This study investigated the protective effects of ginger extract against iron induced oxidative damage in thalassemic RBCs. The pulsed electric field (PEF) technique with high-voltage electric pulses, was used for ginger extraction. The PEF significantly enhanced the yield of bioactive compounds, 6-shogaol and total phenolic content compared to conventional maceration technique. The 6-shogaol-rich ginger extract possessed a potent antioxidant activity by scavenging free radicals (ABTS^•+^ and DPPH^•^), and inhibiting of AAPH-induced RBC hemolysis in both healthy and β-thalassemic RBCs. Additionally, the extract exhibited iron-chelating properties by decreasing non-heme iron levels on the RBC membrane, thereby reducing lipid peroxidation reaction. These findings suggest that 6-shogaol-rich ginger extract processed with PEF could serve as a potential therapeutic agent, exhibiting powerful antioxidant and red blood cell membrane iron-chelating properties to protect against oxidative damage and hemolysis in iron-overloaded β-thalassemia patients.

## 1. Introduction

β-thalassemia is a hereditary hematological disorder that impacts the production of beta-globin chains, an important component of hemoglobin. The reduction of beta-globin chain synthesis disrupts the balance of globin chains, resulting in an accumulation and precipitation of unpaired alpha-globin chains. This imbalance shortens the lifespan of red blood cells (RBC) and increases their hemolysis [1,2]. In severe cases of thalassemia, blood transfusions are necessary to maintain an adequate level of hemoglobin and reduce the risk of anemia. However, regular blood transfusion, along with increased gastrointestinal iron absorption in thalassemia, leads to iron overload [3]. There is no active mechanism to eliminate excess iron from the body; therefore, this iron can accumulate and induce the production of reactive oxygen species (ROS) through the Fenton reaction, consequently causing a damage to biomolecules, including proteins, DNA, and lipids [4,5]. Additionally, the aggregation of alpha-globin chains in beta-thalassemia patients can further induce ROS production, contributing to oxidative stress and accelerating the senescence of RBC [6,7]. The oxidative stress adversely affects the RBC membrane, leading to membrane lipid peroxidation and loss of membrane integrity [8]. Prolonged exposure to oxidative stress causes premature aging of RBC, which increases the susceptibility of RBC to elimination by the spleen, thereby shortening their lifespan [9]. Thus, antioxidant therapy has emerged as a potential strategy to mitigate the oxidative stress associated with RBC hemolysis in the transfusion dependent β-thalassemia (TDT) patients.

Ginger (*Zingiber officinale*) is a medicinal plant that contains various bioactive components that have been reported to possess strong antioxidant and iron chelating activity which consequently impacts the decreasing of ROS and oxidative stress. [10,11]. The bioactive ingredients of ginger have been identified for decades, with phenolic compounds such as gingerols, shogaols, paradols, and zingerone which have been identified as primary non-volatile, pungent compounds. Among these constituents, 6-gingerol and 6-shogaol are the most abundant compounds in ginger extract and exhibit strong biological activity [12]. Shogaol, a dehydrated derivative of gingerol, has been showed to possess various biological activities, including antioxidant, anti-inflammatory, and anti-cancer properties [13]. Additionally, the accumulated evidence has demonstrated that 6-shogaol exhibited more potent antioxidant and anti-inflammatory properties compared to 6-gingerol, likely due to the presence of the α,β-unsaturated ketone moiety in shogaol [14,15]. This study applied the pulsed electric field (PEF) technique to increase the yield of bioactive compounds in ginger extract especially 6-shogaol. PEF has been used to improve extraction yields by inducing the irreversible electroporation of cell membrane, leading to the release of bioactive compounds [16]. The comparison of active compound levels between the pulsed electric field (PEF) method and the conventional method will be performed. Subsequently, we evaluated the protective effects of 6-shogaol-rich ginger extract, obtained through PEF extraction, on iron-induced oxidative stress in red blood cells of iron overloaded β-thalassemia patients. This study focused on assessing antioxidant and iron-chelating properties as a potential natural strategy to extend red blood cell lifespan, and delay hemolysis in iron-overloaded β- thalassemia patients.

## 2. Materials and methods

### 2.1 Plant materials

The fresh ginger was purchased from the Thai Royal Project store located in Chiang Mai, Thailand. The voucher specimen was 0023403 and was deposited at the herbarium of Faculty of Pharmacy, Chiang Mai University, Thailand. The ginger was diced into small pieces and then dried in the oven at 50 °C. The dried ginger was ground into the powder by using a blender. The powder of ginger was kept at −20 °C for further extraction.

### 2.2 Chemical materials

2,2’-Azino-bis 3-ethylbenzothiazoline-6-sulfonic acid (ABTS), dimethyl sulfoxide (DMSO), 2,2-diphenyl-1picrylhydrazyl-hydrate (DPPH), and thiobarbituric acid (TBA) were purchased from Thermo Fisher Scientific, Inc., Waltham, MA, U.S.A. Additionally, 2,2′-azobis 2-amidinopropane (AAPH) and ascorbic acid were purchased from Sigma–Aldrich Inc., St. Louis, MO, U.S.A.

### 2.3 Ginger extract preparation

#### 2.3.1 Macerate extraction.

The ginger powder was mixed in 80% ethanol (ratio 1:10 w/v) and shaken at 200 rpm at 25°C for 24 hours. Then, the mixture was filtered through the Whatman No.1 filter paper. The solvent was subsequently removed from the ginger mixture by rotary evaporator and freeze dryer. The ginger extract was kept at −20 °C for further experiment.

#### 2.3.2 Pulsed Electric Field (PEF) extraction.

The ginger powder was macerated with 95% Ethanol at a ratio of 1:10 (*w/v*) in a PEF treatment chamber at the Department of Electrical Engineering, Faculty of Engineering, Rajamangala University of Technology Lanna, Chiang Mai, Thailand. The stainless-steel PEF treatment chamber, which had a capacity of 300 mL, was installed in the treatment cabinet along with the stainless-steel electrodes. The ginger macerate was subjected to an electric field (10 kV/cm) at a frequency of 5 Hz with a pulse width of 1 µs for 20 min [17]. The ginger mixture was subsequently filtered through the Whatman No.1 filter paper. The solvent was afterward removed from the extract by using a rotary evaporator and freeze-dry technique.

### 2.4 Determination of bioactive compositions in ginger extracts

#### 2.4.1 Measurement of 6-gingerol and 6-shogaol.

Ginger extracts from both methods were analyzed for 6-gingerol and 6-shogaol contents using High-Performance Liquid Chromatography (HPLC). The measurement was conducted according to the procedure described by Chuljerm et al. (2023) [18]. A reverse phase column (C18 type, 250 mm x 4.6 mm, 5 µm pore size, Agilent Technologies, Santa Clara, CA, USA), flow rate of 1.0 mL/min, and wavelength measurement at 230 nm were applied for identifying 6-gingerol and 6-shogaol content. The mobile phase consists of water (A) and acetonitrile (B) with the following gradient elution: 0 min 45% B, 8 min 50% B, 17 min 65% B, 32 min 100% B, 38 min 100% B, 43 min 45% B, 48 min 45% B. The 6-gingerol and 6-shogaol in ginger extract were identified based on the specific retention time and the concentration was calculated from the standard of 6-gingerol and 6-shogaol.

#### 2.4.2 Measurement of total phenolic content.

The total phenolic content (TPC) of the ginger extracts was measured using the colorimetric Folin–Ciocalteu method as described previously by Paradee et al. (2019) [19]. Briefly, 20 µL of ginger extract and gallic acid were mixed with 80 µL of Folin–Ciocalteu reagent and 100 µL of 7.5% sodium carbonate. The reaction mixture was incubated at room temperature in the dark for 2 h. The absorbance was monitored at 765 nm using a UV-Vis spectrophotometer microplate reader. The TPC of ginger extract was calculated based on the standard curve of gallic acid and expressed as milligrams of gallic acid equivalents (GAEs) per gram extract.

#### 2.4.3 Measurement of total flavonoid content.

The total flavonoid content (TFC) of the ginger extracts was determined using the aluminum chloride colorimetric method [19]. In brief, 20 µL of the ginger extract and Quercetin were mixed with 80 µL of 0.5% aluminum chloride solution, and 100 µL of 40 mM potassium acetate solution. The reaction mixture was incubated at room temperature in the dark for 30 min. The absorbance was monitored at 415 nm using a UV-Vis spectrophotometer microplate reader. The TFC was calculated by constructing the standard curve of Quercetin and expressed as milligrams of quercetin equivalent (QE) per gram extract.

### 2.5 Determination of the radical scavenging activity

#### 2.5.1 (2,2’-Azinobis 3-ethylbenzothiazoline-6-sulphonate) ABTS radical scavenging assay.

The antioxidant potential of the ginger extract was analyzed using the ABTS assay [20]. Initially, the ABTS radical cation (ABTS^•+^) was produced by mixing 7 mM ABTS solution and 2.45 mM potassium persulfate solution at room temperature in the dark for 12–16 h. The ABTS^•+^ mixture solution was diluted with ethanol to achieve the absorbance of 0.7 ± 0.1 at 750 nm. In this study, 20 µL of ginger extract and Trolox solution were incubated with 180 µL of ABTS^•+^ solution at room temperature for 5 min. The absorbance at 750 nm was monitored using UV-Vis spectrophotometer microplate reader. The percent inhibition of ABTS^•+^ radical was calculated from the following formula: Inhibition of ABTS^•+^ production (%) = ((A – B)/A) × 100, where A is the absorbance of the control and B is the absorbance of the sample; the ABTS^•+^ radical scavenging activity of ginger extract and Trolox was expressed as half maximal inhibitory concentration (IC50), calculated from the graph plotted between the percentage of inhibition and the concentration of the compound.

#### 2.5.2 (2,2’-Diphenyl-1-picrylhydrazyl-hydrate) DPPH radical scavenging assay.

The radical scavenging activity of ginger extract and Trolox was determined by DPPH assay [20]. Briefly, 20 µL of ginger extract and Trolox solution were mixed with 180 µL of 176 µM DPPH solution. The mixture solution was incubated at room temperature in the dark for 30 min. The absorbance was monitored at 520 nm using UV-Vis spectrophotometer microplate reader. The percent inhibition of DPPH radical by ginger extract and Trolox was calculated using the following formula: Inhibition of DPPH• production (%) = ((A − B)/A) × 100, where A is the absorbance of the control and B is the absorbance of the sample; the DPPH radical scavenging activity of ginger extract and Trolox was express as IC50 which determined from the graph plotted between the percentage of inhibition and the concentration of the compound.

### 2.6 Determination of anti-hemolysis activity

#### 2.6.1 Ethical approval.

The protocol for anti-hemolysis, red blood cell non-heme iron, and TBARS measurement which used normal human red blood cells, was approved by Research Ethics Committee of Faculty of Medicine, Chiang Mai University (Study Code: BIO-2564–08002). The thalassemia patient’s blood was performed in accordance with the Declaration of Helsinki and approved by the Research Ethical Committee for Human Study of Faculty of Medicine, Chiang Mai University, Chiang Mai, Thailand (Study code: MED − 2561 − 05846) regarding the thalassemia control group without an intervention of the co-project.

#### 2.6.2 Subject preparation.

This study was conducted and followed the protocol of approval project (Study Code: BIO-2564–08002 and MED − 2561 − 05846). The first enrollment was performed on 1^st^ November, 2021 and last visit was on 3^rd^ February, 2022. The blood collection was staged at the Adult Thalassemia Clinic, Maharaj Nakorn Chiang Mai Hospital, Faculty of Medicine, Chiang Mai University, Chiang Mai, Thailand. The criteria for thalassemia subjects were Thai adult transfusion dependent β-thalassemia (TDT) patients, aged 20–65 years old, who visited the clinic for regular transfusions, and diagnosed as iron overload with plasma ferritin levels more than 500 µg/L. The healthy subjects were Thai adults who were age- and sex-matched with β-thalassemia (TDT) patients and did not take any additional supplement.

#### 2.6.3 Anti-hemolysis activity in red blood cells derived from β-thalassemia patients.

The biological activity of ginger extract on red blood cell anti-hemolysis activity was determined by using blood samples collected from healthy persons and β-TDT patients [21]. The blood samples were centrifuged at 4 °C, 3,000 g for 10 min. The red blood cells were then isolated and washed with PBS several times. After washing, the red blood cells were resuspended in phosphate buffer solution (PBS) pH 7.4 to prepare 10% red blood cell suspension. In the reaction, 100 µL of 10% red blood cell suspension was incubated with 100 µL of ginger extracts or ascorbic acid at 37 °C for 20 min with gentle shaking. The reaction mixture was then mixed with 100 µL of AAPH solution and incubated at 37 °C for 2 h with gentle shaking. After incubation, 400 µL of PBS was added and followed by centrifugation at room temperature, 3,000 g for 10 min. The supernatant was collected and monitored photometrically at 540 nm using a UV-Vis spectrophotometer microplate reader.

### 2.7 Iron chelation activity through red blood cell non-heme iron

#### 2.7.1 Preparation of thalassemia erythrocyte ghosts.

The thalassemia erythrocyte ghost was prepared according to the procedure described by Settakorn et.al (2024) [21]. The blood sample of β-TDT patients with iron overload was collected. The blood was centrifuged at 1,500 g for 20 min, and the plasma and buffy coat were removed by aspiration. The packed red blood cells were washed with 0.85% normal saline solution (NSS) several times. Subsequently, 20% red blood cell suspension was prepared. The various concentrations of ginger extract and ascorbic acid were then incubated with 20% red blood cell suspension for 1 h at 37 °C. The mixture was centrifuged at 4°C, 3,000 g for 5 min. The red blood cells were subsequently lysed by adding 0.1% Triton X to obtain only the red blood cell membrane called ghost cells. The red blood cell ghosts were washed several times and resuspended in 10 mM MOPs buffer. The ghost cell solution was stored at 4 °C for further experiments.

#### 2.7.2 Protein determination.

The concentration of protein in the ghost cell solution was measured using the Bradford assay. Briefly, 10 µL of ghost cell solution and Bovine Serum Albumin (BSA) standard were mixed with 200 µL of Bradford reagent. The reaction mixture was incubated at room temperature for 10 min. The absorbance was monitored at 595 nm using a UV-Vis spectrophotometer microplate reader. The concentration of protein in the ghost cell solution was determined from the BSA standard curve which was conducted by plotting the concentration against absorbance at 595 nm.

#### 2.7.3 Measurement of red blood cell non-heme iron.

In this study, 100 µL of ghost cell solution and standard ferrous ammonium sulfate (FAS) were mixed with 250 µL of 0.6% sodium dodecyl sulfate. Then, 100 µL of the solution mixture was transferred and incubated with 125 µL of reactant solution (0.2% ascorbic acid and 0.2% sodium dithionite in 2 M sodium acetate) at room temperature for 5 min. The mixture was then incubated with 25 µL of color-developing solution (0.4% ferrozine and 2.5% thiourea in deionized water) at room temperature for 2 min. The absorbance of the mixture was monitored at 570 nm using a UV-Vis spectrophotometer [22]. The concentration of non-heme iron was calculated from the FAS standard curve plotted between concentration and absorbance at 570 nm.

### 2.8 Determination of anti-lipid peroxidation activity

Thiobarbituric acid reactive substances (TBARS) result from the lipid peroxidation process. In this study, the method for TBARS measurement was modified from Paradee et al. (2019) [19]. Briefly, 80 µL of ghost solution and standard tocopherol were mixed with 10 µL of 0.2% Butylate hydroxytoluene, 240 µL of 0.44 M Phosphoric acid (H_3_PO_4_), and 160 µL of 0.6% Thiobarbituric acid (TBA) and then the mixture was boiled at 90 °C for 30 min. After cooling, the TBARS products were extracted from the reaction mixture by adding 300 µL of *n*-butanol. The absorbance of the *n*-butanol phase was photometrically monitored at 540 nm using a UV-Vis spectrophotometer. The protein content was measured by using the Bradford assay. The concentration of TBARS was calculated based on the standard curve of 1,1,3,3-tetra methoxy propane (TMP).

### 2.9 Statistical analysis

Data were analyzed using GraphPad Prism version 8.0 (GraphPad Prism Software, San Diego, CA) and expressed as mean ± SD. Statistical significance was analyzed using a one-way analysis of variance with post hoc Tukey-Kramer, in which p < 0.05 was considered a significant difference. The assumptions of normality and homogeneity of variances were evaluated using the built-in diagnostics in GraphPad Prism, and no violations were observed.

## 3. Results

### 3.1 Comparative analysis of the chemical composition in ginger extracts using maceration and PEF techniques

**Fig 1:**
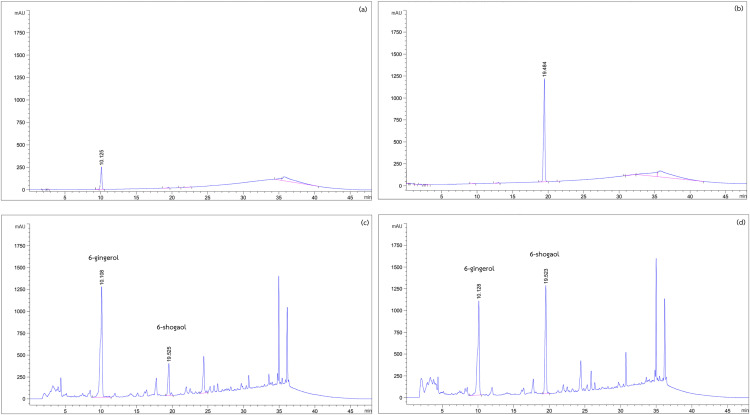
HPLC chromatogram of standard 6-gingerol (0.2 mg/mL) (a), 6-shogaol (0.2 mg/mL) (b), ginger extract obtained by conventional maceration (c), and ginger extract obtained by pulsed electric field extraction (d).

**Fig 2:**
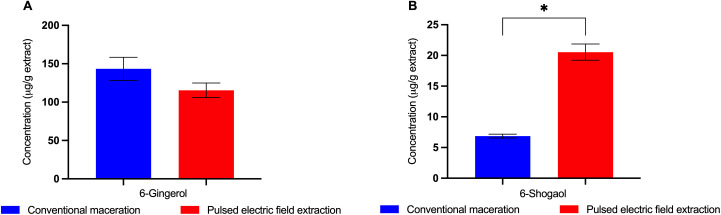
The concentration of 6-gingerol (A) and 6-shogaol (B) in the ginger extract obtained from conventional maceration and pulsed electric field (PEF) extraction. The results were obtained from three independent experiments and expressed as mean ± SD. *P < 0.05 indicates a significant difference between conventional maceration and PEF extraction.

The major phytochemical content in ginger extract, 6-gingerol, and 6-shogaol were identified by high-performance liquid chromatography (HPLC). Figs 1c and 1d show distinct peaks at retention times of 10.108 and 10.128, corresponding to 6-gingerol, and at 19.525 and 19.523, corresponding to 6-shogaol. Both conventional maceration and pulsed electric field (PEF) extraction yielded ginger extract containing 6-gingerol and 6-shogaol. However, PEF extraction resulted in a significantly higher yield of 6-shogaol (20.54 ± 1.32 µg/g extract) compared to conventional maceration (6.86 ± 0.31 µg/g extract), as shown in Fig 2. These findings indicate that PEF extraction effectively enhances the concentration of 6-shogaol in ginger extract. Previous studies have highlighted the potent antioxidant and anti-inflammatory properties of 6-shogaol [15,23]. Therefore, the 6-shogaol-rich ginger extract obtained via PEF extraction was further evaluated for its protective effects against oxidative damage in red blood cells (RBCs).

In accordance with the concentration of 6-gingerol and 6-shogaol, the ginger extract derived from PEF extraction exhibited total phenolic content (TPC) of 122.19 ± 1.52 mg GAE/g extract which is significantly higher than that obtained from the conventional maceration method (115.91 ± 0.55 mg GAE/g extract). Likewise, the level of total flavonoid content (TFC) in ginger extract obtained from PEF extraction was greater than the conventional maceration method, as shown in Table 1.

**Table 1:** Total phenolic content (TPC) and total flavonoid content (TFC) in ginger extract obtained from conventional maceration and pulsed electric field extraction.

Extraction method	TPC (mg GAE/g extract)	TFC (mg QE/ g extract)
Conventional maceration	115.91 ± 0.55	105.71 ± 1.60
PEF extraction	122.19 ± 1.52 *	110.58 ± 4.95

The results were obtained from three independent experiments and expressed as mean ± SD. *P < 0.05 when compared between conventional maceration and PEF extraction.

### 3.2 Radical scavenging activities of 6-shogaol-rich ginger extract

**Fig 3:**
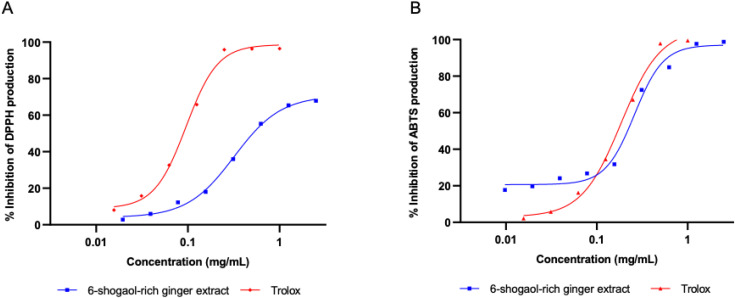
The percentage of DPPH^•^ (A) and ABTS^•+^ (B) inhibition of 6-shogaol-rich ginger extract and Trolox.

The antioxidant activities of 6-shogaol-rich ginger extract obtained from PEF extraction were investigated by examining the ability to scavenge DPPH^•^ and ABTS^•+^ radicals. The results showed that DPPH^•^ radicals were inhibited by 6-shogaol-riched ginger extract and positive control, Trolox with IC50 0.321 ± 0.023 and 0.096 ± 0.004 mg/mL, respectively. Similarly, ABTS^•+^ radicals were scavenged by 6-shogaol-rich ginger extract and Trolox with IC50 0.265 ± 0.012 and 0.186 ± 0.016 mg/mL, respectively (Fig 3).

### 3.3 Anti-hemolysis activity of 6-shogaol-riched ginger extract

**Fig 4:**
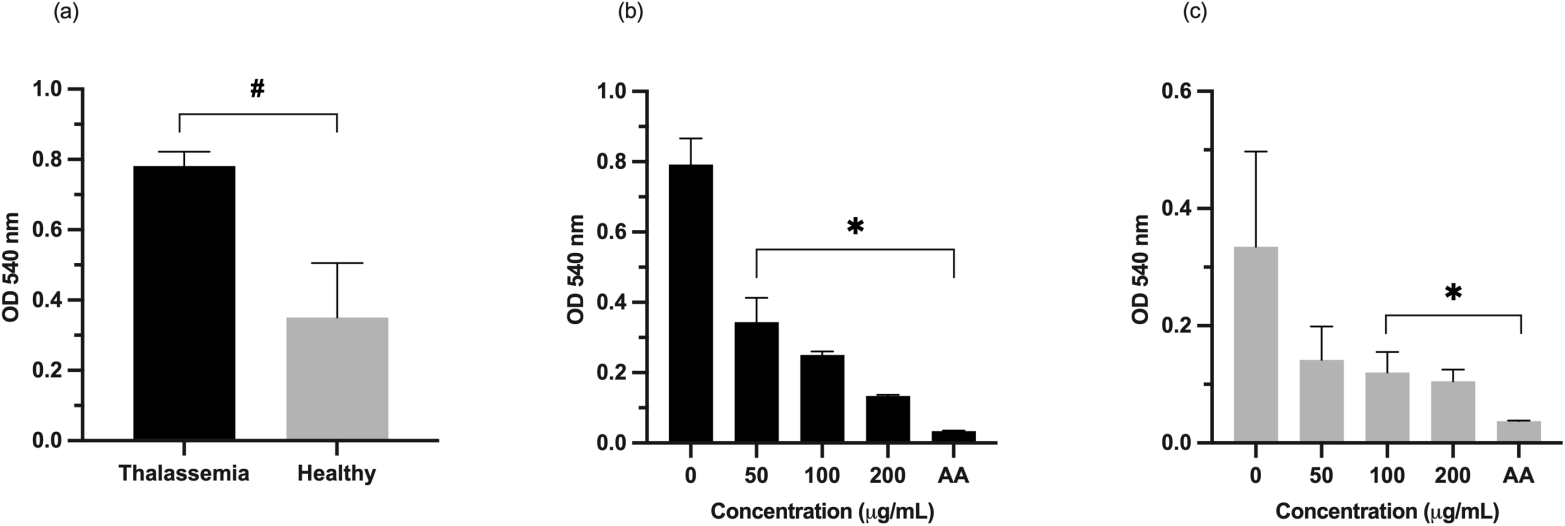
Susceptibility of thalassemic and healthy RBCs to hemolysis (A) and the anti-hemolytic activity of 6-shogaol-rich ginger extract (0–200 µg/mL) compared to ascorbic acid (AA; 100 µg/mL) in thalassemic (B) and healthy RBCs (C). The results were obtained from three independent experiments and expressed as mean ± SD. #P < 0.05 indicates a significant difference between thalassemia patients and healthy individuals. *P < 0.05 indicates a significant difference compared to the untreated group (0 µg/mL).

The susceptibility of RBCs to oxidative damage was assessed using the AAPH assay, which employs an azo compound that generates free radicals capable of inducing oxidative damage to macromolecules such as DNA, proteins, and lipids. RBC membranes are particularly vulnerable to oxidative damage, leading to hemolysis. The results demonstrated that RBCs from thalassemia patients were significantly more prone to hemolysis than those from healthy individuals (Fig 4A).

**Fig 5:**
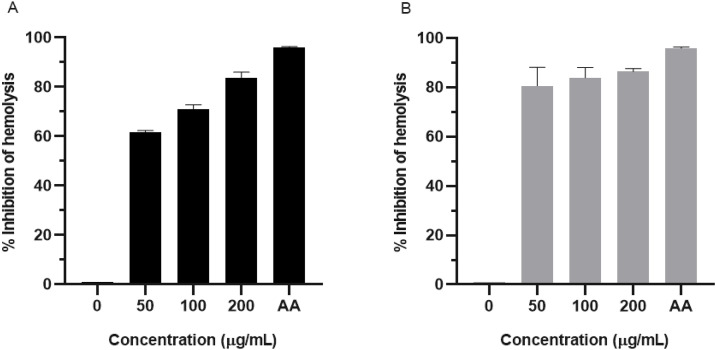
Percentage inhibition of hemolysis by 6-shogaol-rich ginger extract (0–200 µg/mL) and ascorbic acid (AA; 100 µg/mL) in red blood cells obtained from thalassemia patients (A) and healthy individuals (B). The results were obtained from three independent experiments and expressed as mean ± SD.

To evaluate the protective effects of 6-shogaol-rich ginger extract against oxidative damage, RBCs were treated with varying concentrations of the extract (50, 100, and 200 µg/mL) alongside a standard antioxidant, ascorbic acid (AA; 100 µg/mL). The findings revealed that 6-shogaol-rich ginger extract exhibited dose-dependent anti-hemolytic activity in thalassemic RBCs (Fig 4B) and effectively inhibited oxidative damage-induced hemolysis (Fig 5A). A similar protective effect was observed in RBCs from healthy individuals, emphasizing the broad antioxidant activity of the extract (Figs 4C and 5B).

### 3.4 Anti-lipid peroxidation activity of ginger extract

**Fig 6:**
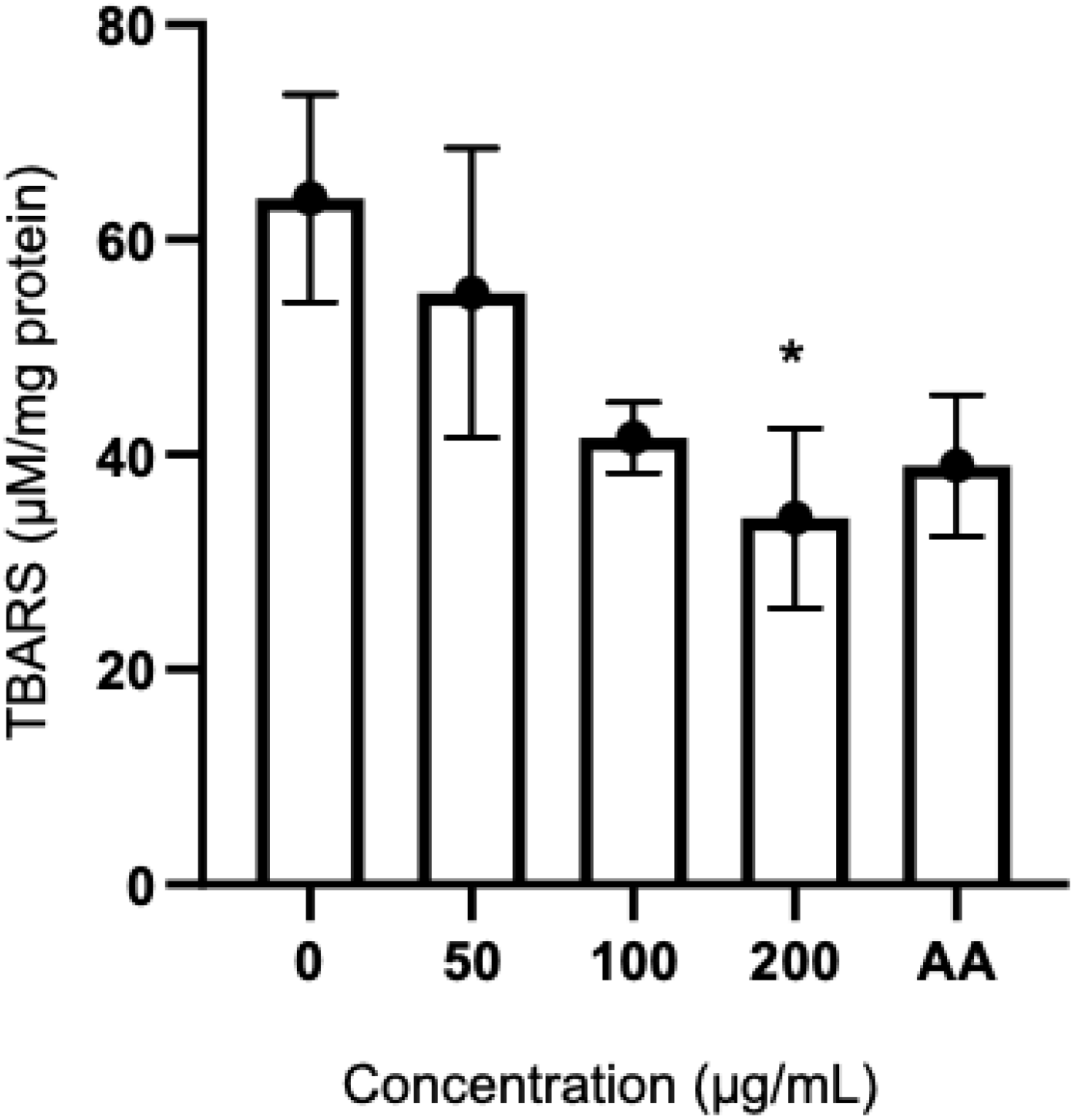
The levels of TBARS product in thalassemia red blood cells treated with 6-shogaol-rich ginger extract at concentrations of 50, 100, and 200 µg/mL and ascorbic acid (AA) at concentration 100 µg/mL. The results were obtained from three independent experiments and expressed as mean ± SD. *P < 0.05 when compared to no treatment group (0 µg/mL).

Free radicals can initiate lipid peroxidation of the cell membrane of red blood cells, leading to hemolysis. This study aimed to assess the protective impact of ginger extract, which is rich in 6-shogaol, on the red blood cells of individuals with thalassemia. The levels of thiobarbituric acid reactive substances (TBARS), a product from the lipid peroxidation process, were measured as an indicator of oxidative damage. The findings revealed that the 6-shogaol-rich extract, at concentrations of 50, 100, and 200 µg/mL, as well as, ascorbic acid (AA), effectively reduced the TBARS levels. Notably, the 6-shogaol-rich ginger extract exhibited a dose-dependent reduction in TBARS, with a significant effect observed at a concentration of 200 µg/mL (Fig 6).

### 3.5 Red blood cell non-heme iron

**Fig 7:**
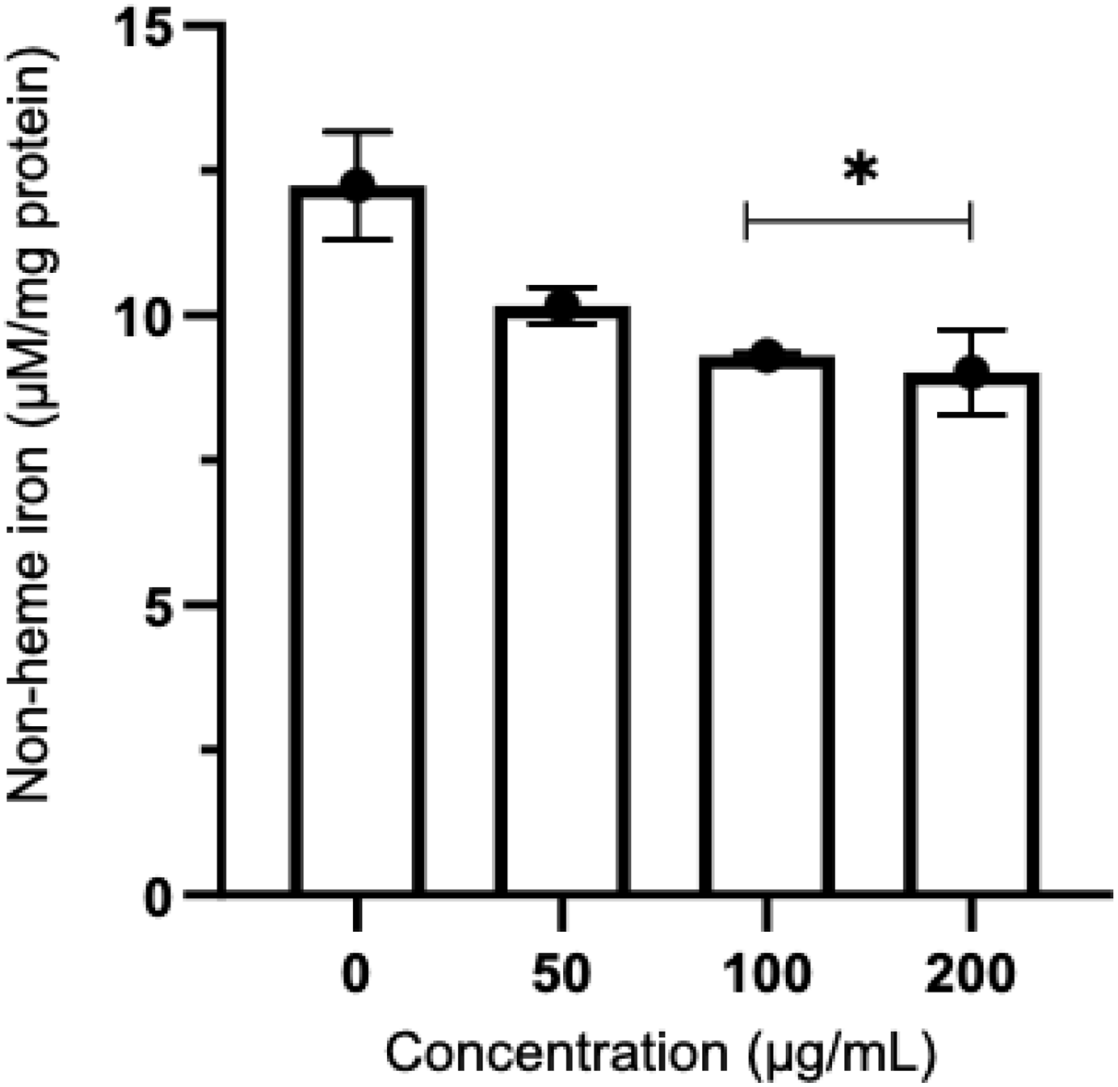
The levels of non-heme iron in thalassemia red blood cell membrane treated with 6-shogaol-rich ginger extract at concentrations of 50, 100, and 200 µg/mL. The results were obtained from three independent experiments and expressed as mean ± SD. *P < 0.05 when compared to no treatment group (0 µg/mL).

The levels of non-heme iron were measured and normalized to the protein content of red blood cell membrane. The finding indicated that treating thalassemia red blood cells with 6-shogaol-rich ginger extract resulted in a reduction of non-heme iron levels. This reduction was dose-dependent and particularly significant at concentrations of 100 and 200 µg/mL (Fig 7).

## 4. Discussion

The PEF extraction technique applies high-voltage electric pulses that induce electroporation of cell membranes, thereby facilitating the extraction of intracellular contents such as phenolic compounds [24,25]. Previous studies have revealed that the PEF treatment enhances the extraction efficacy of bioactive compounds from the plant extraction [26,27]. This study found that ginger extract through PEF treatment markedly enhanced the levels of total phenolic content, total flavonoid content, and 6-shogaol, while also reducing the extraction time compared to the conventional maceration technique. Moreover, the previous findings demonstrated that the heating and drying process accelerated the conversion of gingerol to shogaol, which present a strong antioxidant activity [23,28,29]. Although different ethanol concentrations were used in the two extraction methods (95% for PEF and 80% for maceration), which may have contributed to the variations observed in total phenolic and flavonoid contents between the extraction methods. However, the enhanced yield of 6-shogaol in the PEF extract, which was not solely dependent on solvent polarity, supports the efficiency of the PEF technique.

The previous studies showed that gingerol is the major bioactive compound in fresh ginger, while shogaol is naturally converted from gingerol during the drying process and exhibits potent antioxidant activity [30,31]. The presence of α, β-unsaturated ketone moiety in shogaol structure enhances its radical scavenging ability through covalent binding to target proteins, resulting in great potency and efficacy for antioxidant activity [32–34].

Red blood cells (RBCs) are particularly susceptible to oxidative damage due to the presence of polyunsaturated fatty acids (PUFAs) in their membranes and the iron-rich content of hemoglobin. The RBC membrane is composed of phospholipids, cholesterol, and proteins, with PUFAs accounting for approximately 18% of the lipid component [35]. These PUFAs are particularly prone to oxidative damage, which leads to lipid peroxidation within the RBC membrane. Additionally, thalassemia patients tend to have increased iron absorption from the gastrointestinal tract, which further exacerbating the iron overload. This iron overload exceeds capacity of the body to eliminate, resulting in toxic iron deposition in various vital organs and promoting ROS production, contributing significantly to oxidative stress in thalassemia patients. Our findings highlight the critical role of iron overload in promoting oxidative damage to red blood cells, particularly through membrane lipid peroxidation, underscoring the pathological link between iron accumulation and RBC membrane fragility in thalassemia. In addition, the imbalance of globin chains presents in thalassemic RBCs also contribute to ROS production, particularly superoxide radicals [36]. The findings of this study confirmed the heightened vulnerability of RBC from individuals with iron overloaded transfusion dependent β-thalassemia (TDT) to oxidative damage compared to RBCs from healthy individuals. The elevated oxidative stress, which resulting from the imbalance between ROS production and antioxidant defense system, has been identified as a key factor in the development of critical complications, including cardiovascular diseases and type 2 diabetes, in thalassemia patients [37,38]. While multiple endogenous antioxidant systems play a crucial role in protecting against oxidative damage, they are often insufficient to counteract ROS-induced oxidative stress [38]. Accordingly, exogenous antioxidants are required to maintain an adequate antioxidant defense and mitigate oxidative damage. Previous studies have demonstrated that polyphenolic compounds derived from plants exhibit potent antioxidant activity, making them promising candidates for therapeutic intervention [39,40].

Ginger is a potent source of exogenous antioxidant compounds, as its phytochemicals often contain two or more hydroxyl groups attached to an aromatic ring. This structural characteristic contributes to their ability to neutralize unpaired electrons of free radicals and effectively chelate the toxic iron, a key mechanism involved in ROS production [41]. A previous study by our group demonstrated the potent iron-chelating activity of 6-gingerol-rich ginger extract in iron-loaded hepatocellular carcinoma (Huh7) cells [18]. However, the iron-chelating activity of its dehydrated derivative, 6-shogaol, remains largely unexplored. This study demonstrated that 6-shogaol-rich ginger extract exhibits potent iron-chelating activity, effectively reducing non-heme iron levels in the RBC membranes of individuals with thalassemia. This reduction in iron levels subsequently decreases ROS production, leading to a significant reduction in lipid peroxidation within the RBC membrane. These findings highlight the protective effects of 6-shogaol-rich ginger extract on thalassemic RBCs, emphasizing its potential in mitigating oxidative damage and hemolysis. Furthermore, the study underscores the need for additional research into the role of shogaol-rich ginger extract in modulating the antioxidant defense system in thalassemia.

While the findings are promising, some limitations in this study should be considered. First, the extraction methods (PEF vs. conventional maceration) used different ethanol concentrations, which may have influenced the yield of total phenolic and flavonoid content. The variation in solvent concentration could be a confounding factor. Second, the experiments were conducted using isolated RBCs, which may not fully capture the complexity of in vivo conditions. Therefore, key pharmacokinetic factors such as bioavailability, metabolic stability, systemic distribution, and the route of administration were not evaluated. These aspects are critical for the clinical translation of ginger-based interventions. Further in vivo studies are necessary to confirm the efficacy and safety of 6-shogaol-rich ginger extract in a physiological context.

## 5. Conclusion

This study highlights the potential effect of 6-shogaol-rich ginger extract in mitigating oxidative damage in thalassemia RBCs by chelating excess iron, thereby reducing ROS, lipid peroxidation, and hemolysis. The significant enhancement of 6-shogaol content using the pulsed electric field (PEF) technique further underscores its role in boosting the extract’s antioxidant and iron-chelating activities. These findings emphasize the importance of natural antioxidants like 6-shogaol-rich ginger extract in protecting RBCs membrane. However, the current evidence is limited to ex vivo studies. Further in vivo investigations are needed to evaluate the bioavailability, pharmacokinetics, administration routes, and systemic effects before any therapeutic applications can be considered.

## Supporting information

S1 Graphical abstract_6-Shogaol ginger. (JPG)

## Abbreviation:

β-TDT: transfusion dependent beta-thalassemia;
FAS: ferrous ammonium sulfate
PEF: pulsed electric field
PUFAs: polyunsaturated fatty acids
RBC: red blood cells
ROS: reactive oxygen species
TBARS: thiobarbituric acid reactive substances
TFC: Total flavonoid content
TPC: Total phenolic content
