# Supplementary figures and images for: Pulsed electric field (PEF)–processed 6-shogaol-rich ginger extract protects β-Thalassemic red blood cells from iron-induced oxidative stress and hemolysis
Source: PLoS One. 2025 Sep 12;20(9):e0332386. doi: 10.1371/journal.pone.0332386 (PMC12431221; doi:10.1371/journal.pone.0332386)

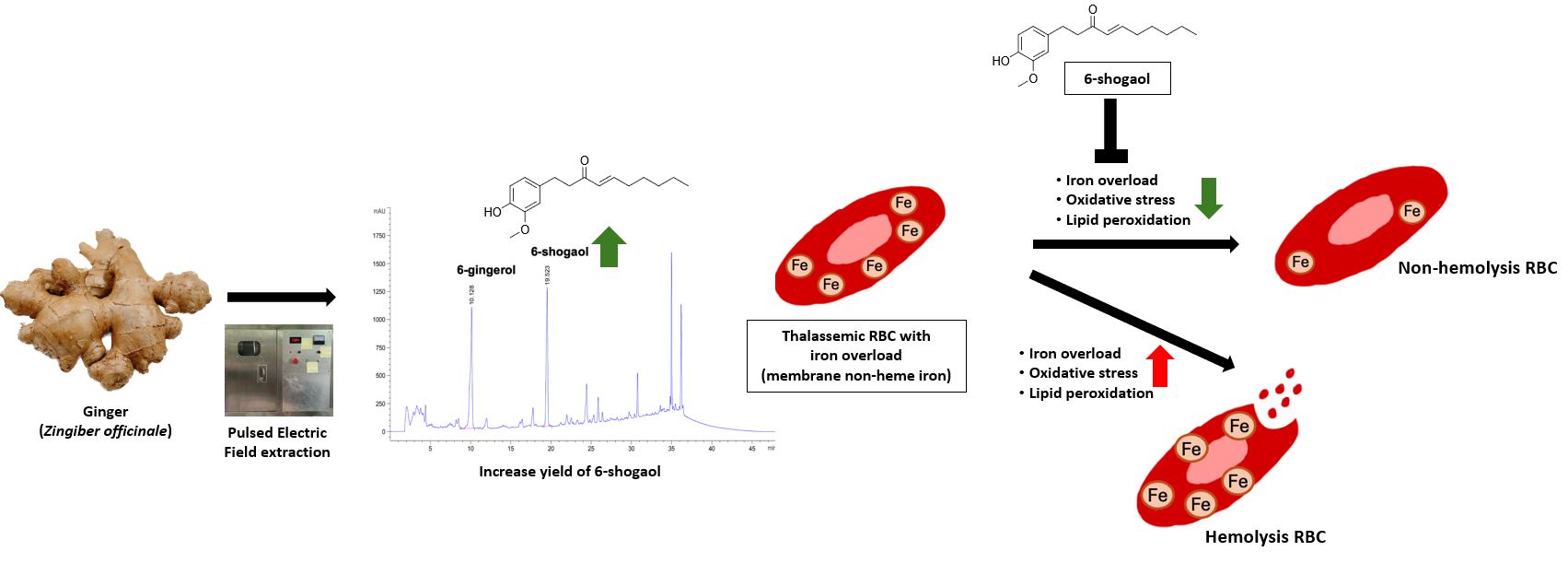

Supplement: S1 — (JPG) [file pone.0332386.s001.JPG]
